# Case report: Successful treatment of a patient undergoing haemodialysis with multifocal hepatocellular carcinoma using atezolizumab and bevacizumab

**DOI:** 10.3389/fonc.2023.1279501

**Published:** 2024-01-04

**Authors:** Shalin Abraham, Adel Samson

**Affiliations:** Leeds Cancer Centre, Leeds Teaching Hospitals National Health Service (NHS) Trust, Leeds, United Kingdom

**Keywords:** hepatocellar carcinoma (HCC), immune check inhibitor (ICI), anti-angiogeneic therapy, atezolizumab, bevacizumab, haemodialysis (HD), end stage kidney disease (ESKD), patient outcome

## Abstract

In the last five years, the advent of combination immune checkpoint inhibitor atezolizumab and anti-angiogenic agent bevacizumab has transformed treatment of unresectable hepatocellular carcinoma. As patient outcomes improve, healthcare professionals will more frequently encounter patients with concomitant hepatocellular cancer and end stage kidney disease on haemodialysis. We present the first case in the literature of a 58-year-old male with multifocal hepatocellular carcinoma undertaking regular haemodialysis who was successfully treated with atezolizumab and bevacizumab with a partial response and stable disease for two years, who suffered grade 1 fatigue, grade 2 hypertension and eventually grade 3 wound infection leading to cessation of bevacizumab. After disease progression on atezolizumab monotherapy, all chemotherapy was stopped. We embed this case in a review of the current literature of atezolizumab and bevacizumab use in patients undertaking haemodialysis and conclude that both targeted therapies may be safely used in these patients. We recommend joint close management of these patients between oncology and nephrology teams, with initial cardiovascular risk stratification before commencing atezolizumab and bevacizumab therapy. During therapy, there should be regular monitoring of blood pressure, or proteinuria if the patient is oliguric under guidance of the dialysis team if preservation of residual renal function is required.

## PART 1 – Treatment of multifocal HCC with atezolizumab and bevacizumab in patient on haemodialysis

We report the case of a 58-year-old male diagnosed with hepatocellular carcinoma (HCC) in 2015 with a background of chronic liver disease and multinodular CP-A5 liver cirrhosis secondary to alcohol excess and chronic Hepatitis B (undetectable DNA) and Hepatitis C infection, which was cured with direct-acting antivirals in 2015. He had a medical history of type 2 diabetes mellitus with retinopathy and a previous diagnosis of glomerulonephritis. He was a smoker of 10 cigarettes a day with no routine activities beyond walking short distances with a stick. He underwent a liver resection in May 2015 of the 30 mm HCC lesion in segment 6 of the liver. Histopathological analysis of the lesion revealed a moderately differentiated hepatocellular carcinoma with no microvascular invasion. Unfortunately, in 2017 the patient developed a new 21 mm lesion in segment 6 and a new 4 mm lesion in segment 4 of the liver. Microwave ablation of the segment 6 lesion took place in June 2017 and follow up computed tomography scan in July 2017 showed a satisfactory response with no active disease or new nodules. Unfortunately, in 2018, there was further disease recurrence with lesions in segment 8 (15mm and 13mm) and segment 3 (8mm). At this point blood tests revealed declining renal function with an EGFR of 42 ml/min and significant proteinuria of 3.7 g/L. Serum alpha-fetoprotein levels remained within normal limits throughout.

**Figure 1:**
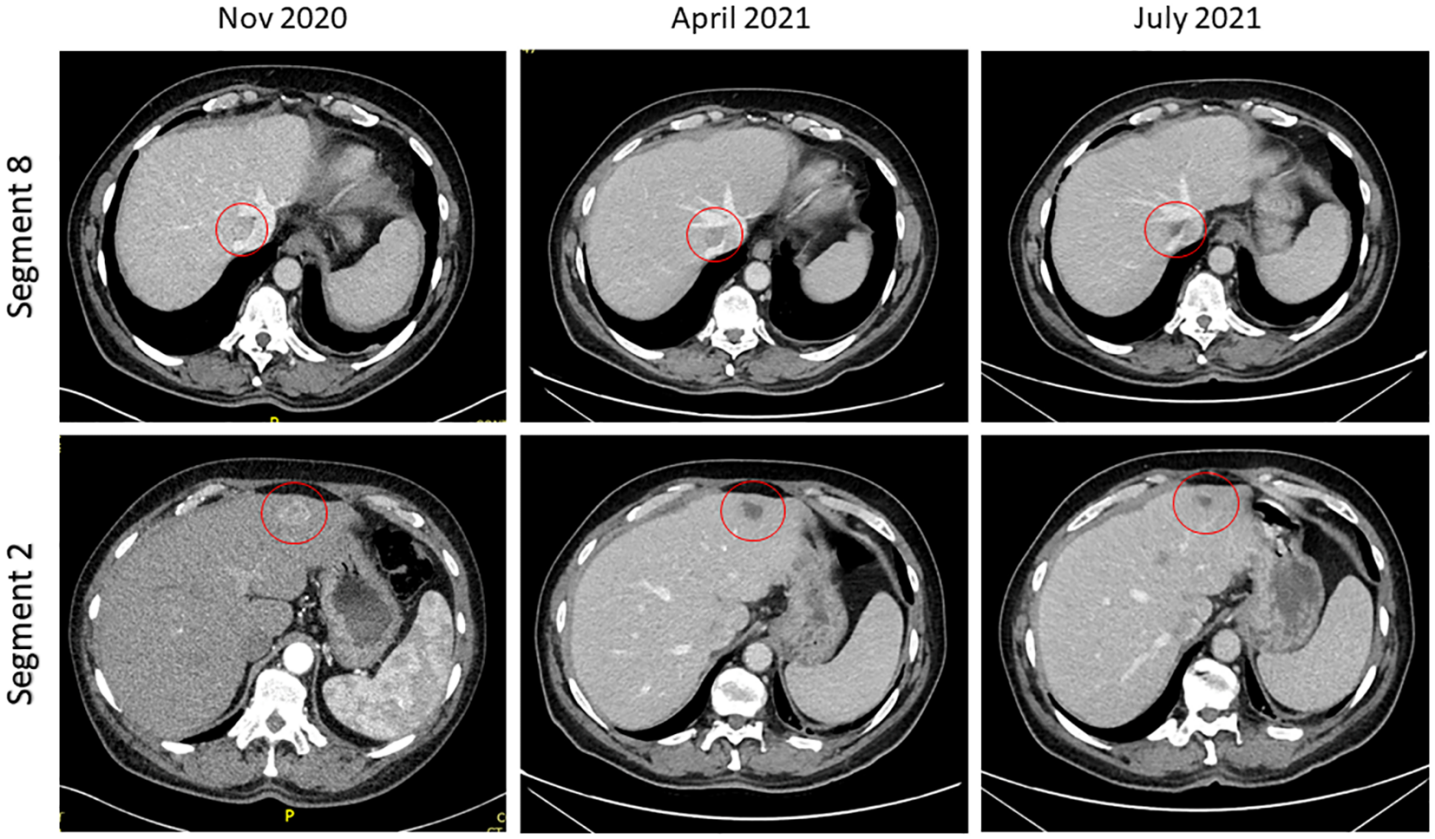
Computed tomography imaging demonstrating response in segment 2 and segment 8 lesions post cycle 4 and post cycle 8 of combination atezolizumab/bevacizumab.

**Figure 2:**
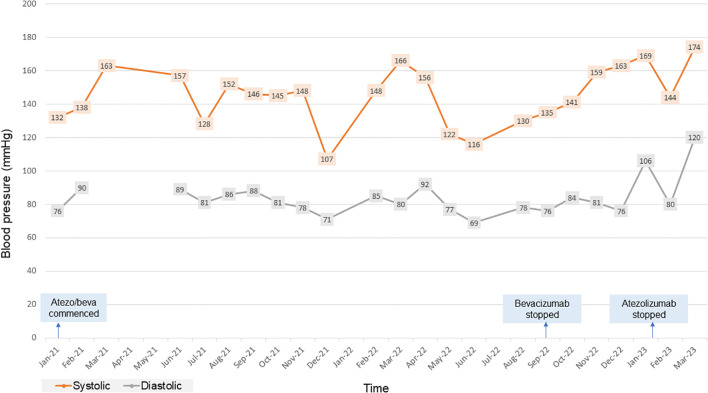
Chart showing the patient’s blood pressure readings over time for the duration of combination atezolizumab/bevacizumab treatment. atezo/beva, atezolizumab/bevacizumab.

**Figure 3:**
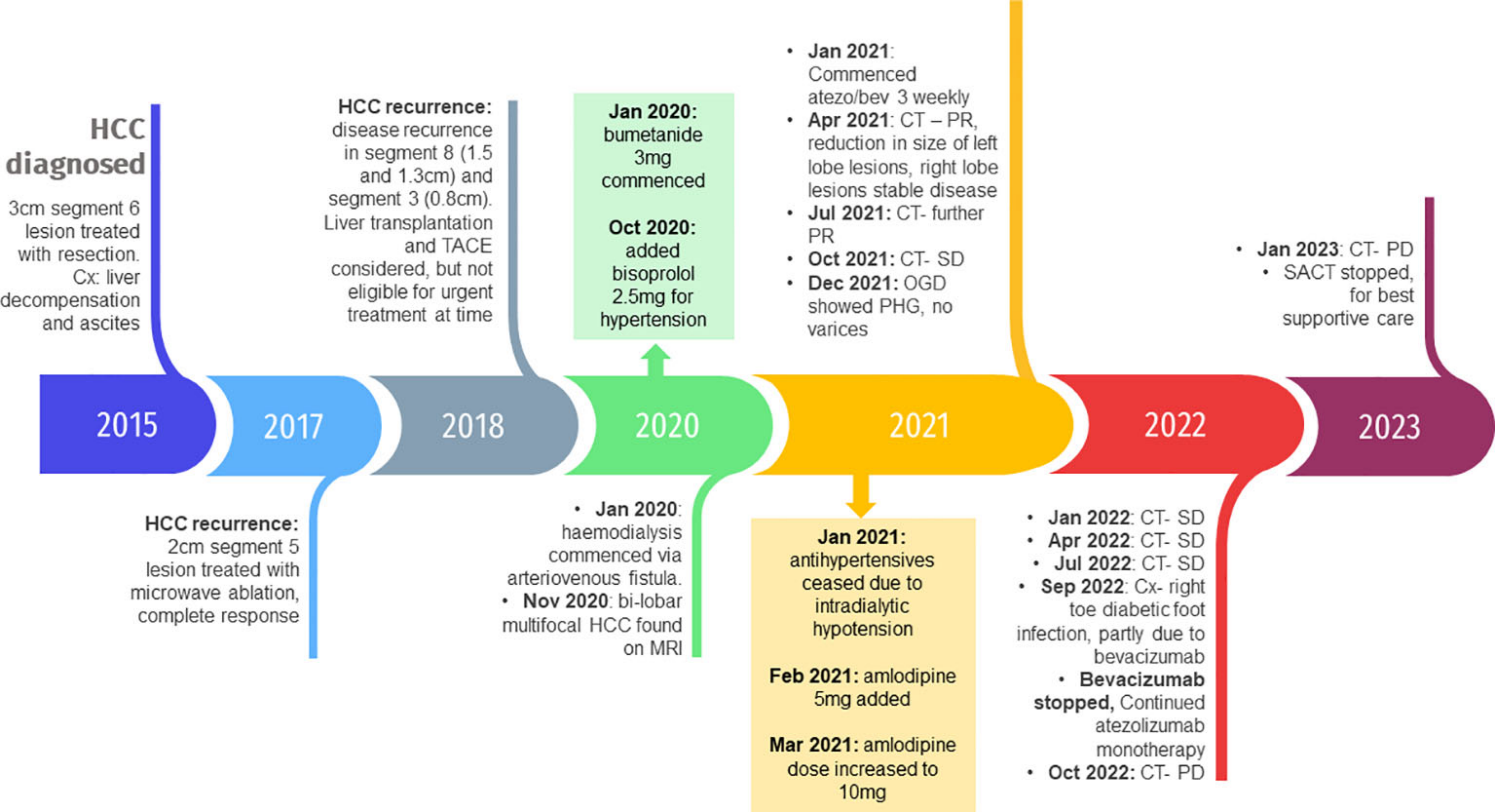
Timeline of the patient’s clinical course with key events, complications and medication changes. HCC, hepatocellular carcinoma; Cx, complications; TACE, transarterial chemoembolization; MRI, magnetic resonance image; CT, computed tomography; PR, partial response; SD, stable disease; OGD, oesophagogastroduodenoscopy; PHG, portal hypertensive gastropathy; PD, disease progression; SACT, systemic anticancer treatment.

In January 2020, the patient was found to have end stage renal failure with refractory fluid retention and biopsy-proven diabetic nephropathy and commenced 3 times a week haemodialysis via an arteriovenous fistula. A further MRI scan of the liver in November 2020 revealed bi-lobar multifocal HCC with over 10 LR5 tumours. Marker lesions in segment 2 (26mm and 13mm) and segment 8 (19mm) were identified. In January 2021 after a year of haemodialysis, the patient commenced treatment with 1200mg atezolizumab and 1200mg bevacizumab (15mg/kg) every 3 weeks. The patient’s medication list included losartan, simvastatin, alfacalcidol, bumetanide, clopidogrel and Humulin M3 insulin. Prior to commencing treatment with atezolizumab and bevacizumab, the patient’s blood pressure was 132/76mmHg. At this point the patient was self-caring and could walk a few hundred yards with a stick, giving him a performance status of 1. Remarkably, the patient tolerated the infusions with grade 1 fatigue according to Common Terminology Criteria for Adverse Events (1). CT scan after 4 cycles showed stable disease with a trend towards response. **Figure 1** shows the trend of the segment 2 lesion and the segment 8 lesion over the time on combination atezolizumab and bevacizumab therapy. The segment 8 lesion responded particularly well, demonstrating a 40% decrease in size from 20 mm to 12 mm between April 2021 and April 2022, with stable disease following this. Blood pressure measurements were made prior to each cycle and as part of haemodialysis monitoring. **Figure 2** shows the blood pressure readings against time throughout the patient’s course. The patient’s antihypertensive regime was managed by both the renal and oncology teams. In 2020, bumetanide and bisoprolol was added by the nephrology team to control fluid overload and blood pressure, however these were ceased in January 2021 due to intradialytic hypotension. In February 2021, amlodipine was restarted by the dialysis team and then doubled by the oncology team in March due to a pre-cycle 4 grade 2 hypertension (1). In March 2022, the patient’s systolic blood pressure again rose above 160mmHg, however after the patient provided home blood pressure readings, no changes were made to the antihypertensive regime. Proteinuria monitoring during bevacizumab treatment was not possible as the patient was anuric. In September 2022 after 27 cycles, bevacizumab was permanently ceased due to grade 3 right toe diabetic foot infection requiring a prolonged course of antibiotics and right hallux and second toe amputation, as it was felt to be contributing to the patient’s peripheral vascular disease and hindrance of wound healing (1). Following cessation of bevacizumab unfortunately the patient experienced progression of disease. Subsequently, atezolizumab has been stopped and the focus of care has shifted to best supportive care. **Figure 3** is a summary infographic of the patient’s clinical timeline.

## Part 2

### Introduction

HCC is the third leading cause of cancer-related deaths worldwide and is an increasing global health challenge as the number of new cases of primary liver cancer is predicted to increase by over 55% by 2040 (2). The systemic treatment of unresectable HCC has transformed over the last 5 years. The pivotal phase 3 trial IMBrave150, published in 2020, showed that the programmed cell death ligand 1 (PD-L1) inhibitor atezolizumab plus vascular endothelial growth factor (VEGF) inhibitor bevacizumab resulted in superior progression-free and overall survival compared to the multikinase inhibitor sorafenib (3).

UK National Institute of Clinical Excellence (NICE) and National Comprehensive Cancer Network (NCCN) guidelines have been updated to reflect this, now recommending atezolizumab plus bevacizumab to treat unresectable HCC in adults who have not had previous systemic treatment in patients with Child- Pugh (CP) grade A liver impairment and Eastern Cooperative Oncology Group (ECOG) performance status of 0 or 1 (4, 5). There is a complex relationship between HCC and end stage kidney disease (ESKD). ESKD is a risk factor for HCC development and vice versa, HCC can cause renal dysfunction, for example through hepatorenal syndrome or direct tumour invasion. Furthermore, there are common risk factors for HCC and ESKD development, and ESKD with dialysis negatively affects HCC prognosis. As patients with advanced HCC experience improved survival with new systemic therapies, clinicians will more frequently encounter HCC patients with co-existing ESKD receiving haemodialysis (6). It is therefore paramount to understand the impact of renal function and dialysis on HCC patients treated with new systemic therapies. We present the first case, to our knowledge, of a 58-year-old male with multifocal HCC and end stage renal failure receiving haemodialysis, who is being successfully treated with combination atezolizumab and bevacizumab. We embed the case within a brief literature review and discuss further important clinical considerations when treating patients with HCC and ESKD on haemodialysis (7).

### Discussion

Atezolizumab and bevacizumab are humanised immunoglobulin antibodies of the IgG1 isotype, with a large molecular size (around 145 kDa). Pharmacokinetically, they have a small volume of distribution, mostly in the vascular compartment. The main route of elimination is intracellular catabolism following receptor-mediated endocytosis and hepatic or renal elimination is negligible. They are too large to be secreted through the glomerular filtration barrier, except possibly in high-grade non-selective proteinuria. Furthermore, the molecular size of these immunoglobulins is larger than dialysis pores, meaning they will not be filtered out of the bloodstream during haemodialysis (8–11). As a result, there is no reason to alter the dosage of either drug or no reason to believe there would be an increase in adverse events due to accumulation. As neither atezolizumab or bevacizumab dialyse, both drugs can be administered before or after haemodialysis (12–14).

**Table 1** shows a summary of current literature of atezolizumab use in patients with ESKD. Atezolizumab at full dose has been used successfully in multiple case reports of patients with dialysis. Data from a large real-world database of patients receiving PD-1 inhibitors for lung, renal, bladder, head and neck or melanoma, showed no increased rate of immune-related adverse events in patients with ESKD versus those without (20). Whilst immune checkpoint inhibitors can be given in patients with ESKD receiving dialysis, important consideration must be given to patients with previous kidney transplants as immune checkpoint inhibition can precipitate transplant rejection and allograft failure (15). Furthermore, in all patients there is a need to monitor renal function as immune checkpoint inhibition can cause a decline in renal function, a phenomenon referred to as immune checkpoint inhibitor associated acute kidney injury (ICP-I AKI). A real-world study of 1843 patients estimated incidence of this to be 3% (21).

**Table 1:** Summary of current literature: atezolizumab.

Author	Primary tumour	Anti-cancer regimen	Dialysis type and vintage	Side effects	Outcome
Cheun et al, 2019 (9)	68M, Urothelial cell cancer, ECOG PS 2	**Atezolizumab**, 1200mg/body 3 weekly	Haemodialysis, Dialysis vintage 9 days	None	Disease progression, death
Parisi et al, 2019 (6)	Age unknown, Urothelial cell cancer, ECOG PS 2	**Atezolizumab,** 1200mg/body 3 weekly, 13 months	Haemodialysis, Dialysis vintage 7 months	Grade 1 itching, asthenia, nausea, dysgeusia, constipation	Disease progression
Hirsch et al, 2020 (15)	83M, Urothelial cell cancer, ECOG status unknown	**Atezolizumab**	Haemodialysis, Dialysis vintage 60 months	None	Disease stabilisation
Kuo et al, 2020 (16)	58M Urothelial cell cancer, ECOG N/A	**Atezolizumab** **Paclitaxel**	Haemodialysis	Grade 4 neutropaenia Grade 3 anaemia Grade 1 thrombocytopaenia, hepatitis, anorexia, fatigue	Disease progression
	45M Urothelial cell cancer, ECOG N/A	**Atezolizumab** **Paclitaxel**	Haemodialysis	Grade 4 anaemia	Disease stabilisation
	66M Urothelial cell cancer, ECOG N/A	**Atezolizumab**	Haemodialysis	Grade 4 neutropaenia, anaemia, toxic epidermal necrolysis Grade 3 TB Peritonitis grade 1 thrombocytopaenia	Partial response
Imaji et al, 2021 (17)	80M Small cell lung cancer ECOG PS1	CBCDA 125 mg/body (day 1) Etoposide 40 mg/m2 (days 1, 2, and 3) **Atezolizumab** 1200 mg/body (day 1)	Haemodialysis, Dialysis vintage 7 years	Grade 1 thrombocytopaenia, grade 4 neutropaenia and grade 4 leukopaenia	Partial response
Watari et al, 2021 (18)	69M Small cell lung cancer ECOG PS0	**Atezolizumab** 1200 mg/body on day 1 VP‐16 (50 mg/m2) on days 1 and 3, CBDCA (300 mg/m2) on day 1	Dialysis- type NA	Grade 3 neutropaenia, grade 4 thrombocytopaenia Management: Carboplatin and etoposide dose reduction	Disease progression
	73M Small cell lung cancer ECOG PS1	**Atezolizumab** 1200 mg/body on day 1 VP‐16 (40 mg/m2) on days 1 and 3, CBDCA (240 mg/m2) on day 1, 210mg/m2 from cycle 2	Dialysis- type NA	Grade 3 neutropaenia	Partial response
Imai et al, 2023 (19)	70-79M Large cell neuroendocrine carcinoma ECOG PS1	Carboplatin at a dose of 240 mg/m2 on day 1, etoposide at a dose of 40 mg/m2 on day 1 and 3, **atezolizumab** 1200 mg/body on day 1	Haemodialysis	Anaemia (grade unknown) leading to grade 3 heart failure	Partial response

**Table 2** shows a summary of current literature of bevacizumab use in patients with ESKD. Renal toxicity, particularly proteinuria and hypertension are seen in use of these agents. VEGF and its receptors are expressed in abundance in podocytes, glomerular and tubular cells in the kidney and are crucial in the survival of mesangial and endothelial cells and therefore to the integrity of endothelial fenestrations in the glomerular filtration barrier. Proteinuria is a known dose-dependent adverse effect of bevacizumab and is due to VEGF-inhibition induced structural changes in glomerular cells; dysregulation of the kidney repair process and increased glomerulosclerosis; and reduction in endothelial fenestrations and loss of selective glomerular permeability (29–31). As therapeutic monoclonal antibodies are large molecules; they are not typically cleared by renal or dialysis filtration. However, it is possible that high-grade non-selective proteinuria can result in renal clearance of monoclonal antibodies (32). Current guidelines recommend suspending bevacizumab administration if 24-hour urine-protein collection is >2g and treatment discontinuation in cases of nephrotic-range proteinuria (>3.5g), which reduces the risk of any possible renal clearance and altered pharmacokinetics of either atezolizumab or bevacizumab (33). In our patient, proteinuria was not routinely monitored as he was anuric. In patients who are oliguric while on haemodialysis, whether strict proteinuria monitoring is needed is unclear. Proteinuria is a marker of ESKD progression and an independent risk factor for cardiovascular mortality (34, 35). Higher degrees of proteinuria in chronic haemodialysis patients are associated with inflammatory and cardiovascular markers of disease (36). Preservation of residual renal function in a longitudinal study of over 6000 patients was associated with better patient survival (37). Therefore, in oliguric patients undergoing haemodialysis, we recommend proteinuria monitoring in partnership with the nephrology team, to minimise the impact that bevacizumab-induced proteinuria has on ESKD progression and to help preserve residual renal function.

**Table 2:** Summary of current literature: bevacizumab.

Author	Primary tumour	Anti-cancer regimen	Dialysis type and vintage	Side effects	Outcome
Garnier-Viougeat et al, 2006 (12)	23M Metastatic renal cell cancer, ECOG N/A	**Bevacizumab** 5mg/kg every 2 weeks	Haemodialysis Dialysis vintage unknown	N/A	N/A
Inauen et al, 2007 (22)	48F Metastatic colorectal cancer, ECOG N/A	**Bevacizumab** 5mg/kg every 2 weeks and Cetuximab (400mg/m2 for first dose then 250mg/m2 weekly)	Haemodialysis Dialysis vintage unknown	Fatigue, dry skin	Disease progression
Izzedine et al, 2009 (13)	23M Metastatic renal cell cancer, ECOG N/A	IL-2–interferon-alfa cytokine protocol and a 6-month period of 5 mg/kg of **bevacizumab** every 2 weeks, replaced by sunitinib once daily (50 mg/day) orally for 4 weeks every 6 weeks following PD	Haemodialysis Dialysis vintage unknown	N/A	Disease progression
Horimatsu et al, 2011 (23)	50M Metastatic colorectal cancer, ECOG N/A	MFOLFOX 6 plus **bevacizumab** every 3 weeks, oxaliplatin titrated up to 85mg/m2	Haemodialysis Dialysis vintage 5 years	Grade 1 peripheral neuropathy	N/A
Syrios et al, 2013 (24)	50F Metastatic renal cell cancer, ECOG N/A	Interferon alfa-2b 6 MU three times per week, **Bevacizumab** 200 mg weekly	Haemodialysis Dialysis vintage unknown	Grade 4 haemorrhagic gastritis secondary angiodysplasia, necessitation cessation of bevacizumab	Partial response
Shetty et al, 2014 (25)	3 patients with renal cell carcinoma Sex, age unknown ECOG N/A	10mg/kg **Bevacizumab** every 2 weeks	Haemodialysis Dialysis vintage unknown	Grade 1 and grade 2 fatigue, nausea and hypertension exacerbation	N/A
Van Berlo-van de Laar et al, 2018 (26)	77M Metastatic rectal cancer, ECOG N/A	FOLFOX and **bevacizumab** (oxaliplatin 70 mg/m2, folinic acid 200 mg/m2, 5-FU 340 mg/m2 bolus and 2040 mg/m2 continuous infusion for 44 hours and bevacizumab 5 mg/kg) every three weeks	Haemodialysis Dialysis vintage 1 year	None	Disease progression
Funasaka et al, 2019 (27)	65M Metastatic colon cancer, ECOG PS 1	MFOLFOX-6 L-OHP: 60 mg/m2, l-LV: 200 mg/m2, bolus 5-FU: 400 mg/m2, 46-hr injection of 5-FU: 2,400 mg/m2 plus **bevacizumab** 5 mg/kg	Haemodialysis Dialysis vintage 9 months	Grade 1 peripheral neuropathy and grade 2 thrombocytopenia	Partial response
	71M Metastatic rectal cancer, ECOG PS 0	MFOLFOX-6 L-OHP: 60 mg/m2, l-LV: 200 mg/m2, bolus 5-FU: 400 mg/m2, 46-h injection of 5-FU: 2,400 mg/m2 plus **bevacizumab** 5 mg/kg	Haemodialysis Dialysis vintage 15 months	Grade 4 bone marrow suppression, necessitating a 5-FU dose reduction from the second treatment course and a decrease in the chemotherapy frequency to every 4 weeks	Disease stabilisation
	71F Metastatic colon cancer, ECOG PS 2	MFOLFOX-6 L-OHP: 60 mg/m2, l-LV: 200 mg/m2, bolus 5-FU: 400 mg/m2, 46-hr injection of 5-FU: 2,400 mg/m2 plus **bevacizumab** 5 mg/kg	Haemodialysis Dialysis vintage 10 months	Grade 4 neutropenia, grade 3 thrombocytopenia, and grade 1 fatigue FOLFOX therapy was discontinued, and irinotecan/5-fluorouracil/leucovorin (FOLFIRI) plus bevacizumab therapy was initiated as a second-line treatment.	Disease progression
Tanaka et al, 2022 (28)	74M Sigmoid cancer, ECOG PS 0	Irinotecan 120 mg/m2, with levofolinate 200 mg/m2, followed by 5-FU 2400 mg/m2 plus **bevacizumab** 5 mg/kg every 2 weeks	Haemodialysis Dialysis vintage 2-3 years	After cycle 5 grade 4 neutropenia requiring dose reduction of 5-FU and irinotecan, but no bevacizumab related AEs observed	Disease progression

N/A, Not available.

Bevacizumab induced hypertension of all grades has been observed in up to 36% of patients treated with bevacizumab. It is frequently observed in the first cycle of therapy and, like proteinuria, appears to be dose dependent (38, 39). Current recommendation is that bevacizumab can be started if blood pressure is <160/100 mmHg. If during therapy, blood pressure rises by >20 mmHg systolic or 10 mmHg diastolic or rises to >160/100 mmHg, it is recommended to omit a dose and reassess. If blood pressure remains above >150/95 mmHg with ambulatory or home blood pressure monitoring, antihypertensive treatment should commence (40). Hypertension is caused by VEGF-inhibition induced apoptosis and altered rarefaction of vascular endothelial cells and reduced production of vasodilators such as nitric oxide and prostacyclin (29–31). VEGF is also expressed in renal endothelial cells and podocytes, where it maintains normal glomerular filtration rate. VEGF blockade in the kidneys leads to renal injury, activation of the renin-angiotensin system, inadequate renal sodium excretion and volume overload (41, 42). Pre-existing hypertension, increased age, BMI, diabetes and dyslipidaemia are risk factors for developing treatment related hypertension (38, 43). The mechanisms of hypertension in dialysis patients are complex but include: an increase in extracellular body water which can be corrected by increasing ultrafiltration or more frequent dialysis sessions, as well as activation of the renin-angiotensin-aldosterone system; sympathetic over-activity; increased arterial stiffness related to altered calcium and phosphate metabolism; and endothelial dysfuction (44). Hypertension can affect up to 80-90% of dialysis patients (45). Heerspink et al. (2009) showed that blood pressure reduction in dialysis patients with antihypertensive treatment led to a 29% decreased risk of cardiovascular events (relative risk [RR], 0.71, 95% confidence interval 0.55–0.92), and a 20% decreased risk for all-cause mortality (RR, 0.80, 95% confidence interval 0.66–0.96) (46). Interdialytic blood pressure monitoring is gold standard for diagnosing hypertension in haemodialysis patients which can be obtained through ambulatory or home blood pressure monitoring. Strategies for lowering blood pressure include adjusting target dialytic weight and dietary advice regarding salt and fluid restriction in high volume states. In terms of pharmacological treatment, beta-blockers are effective in dialysis patients with left ventricular hypertrophy due to sympathetic overactivity. Dihydropyridine calcium-channel blockers are also effective in lowering blood pressure in high volume states and have great potency in reducing arterial smooth muscle cell contractility in the blood vessels, which is one of the mechanisms associated with VEGF induced hypertension. ACE-inhibitors or angiotensin receptor blockers are also effective; however no study has demonstrated superiority of these agents as anti-hypertensive agents in dialysis patients (47, 48). In a case series by Shetty et al, 2 of 3 of the patients taking 10mg/kg bevacizumab experienced grade 1 and 2 exacerbation of hypertension (25). In our case, the patient was at high risk of experiencing treatment related hypertension due to high BMI and diabetes. He experienced stage 2 hypertension (blood pressure >160/100mmHg), however this was not clearly due to bevacizumab therapy. As seen by **Figure 2**, blood pressure was highly variable and may have been due to variations in total body fluid with dialysis. Nevertheless, blood pressure was jointly managed between oncology and nephrology and when blood pressure rose above 160/100mmHg, home blood pressure monitoring was encouraged, and consequently there was titration of the dose of amlodipine, dietary advice was given and the dialysis regime was altered by the nephrology team.

Unfortunately, the patient in our case progressed while on single agent atezolizumab after bevacizumab was ceased. Currently there are no approved second-line treatments after failure of atezolizumab and bevacizumab. Sorafenib or lenvatinib may be used, but should be used with caution in dialysis patients due to possibility of greater incidence of adverse effects (49). A meta-analysis of possible second line treatments in 2022 analysed 14 phase two or three trials and showed that multikinase inhibitors regorafenib (hazard ratio 0.63, 95% confidence interval 0.50-0.79) and cabozatanib (hazard ratio 0.76, 95% confidence interval 0.63-0.92) significantly prolonged overall survival compared to placebo, after failure of sorafenib therapy (50). It is clear that head-to-head trials of multikinase inhibitors in the second line setting after failure of atezolizumab/bevacizumab therapy are urgently needed.

### Conclusion

We present the first case in the literature of atezolizumab and bevacizumab used together in a patient with multifocal hepatocellular carcinoma and ESKD on haemodialysis. The case report uses doses of both agents comparative to or greater than other cases in the current literature. The patient in our case experienced grade 1 fatigue and grade 2 hypertension, neither of which were dose-limiting toxicities. Unfortunately, bevacizumab was ceased due to a grade 3 wound infection. When bevacizumab was ceased, there was disease progression, clearly demonstrating the synergistic efficacy of combination PD-L1 and VEGF inhibition (51). Haemodialysis should not be a contraindication to commencing these therapies as the literature supports normal pharmacokinetics in haemodialysis patients with both agents. Whilst both targeted therapies are associated with direct renal toxicities, they are less relevant in patients on haemodialysis. Of more relevance are toxicities that could be magnified by use of these agents in ESKD, such as bevacizumab-associated hypertension. We would recommend close joint management of these patients between oncology and nephrology teams and risk stratification of patients for the development of toxicities before commencement. We recommend monitoring of proteinuria if the patient is oliguric and monitoring of hypertension with ambulatory or home blood pressure monitoring, so that they can be appropriately managed to avoid dose-limiting toxicity or periods of cessation that may impact on cancer outcomes.

## Data availability statement

The original contributions presented in the study are included in the article/supplementary material. Further inquiries can be directed to the corresponding author.

## Ethics statement

Written informed consent was obtained from the individual(s) for the publication of any potentially identifiable images or data included in this article.

## Author contributions

SA: Data curation, Software, Writing – original draft. AS: Conceptualization, Funding acquisition, Resources, Supervision, Writing – review & editing.
